# Nitrogen-doped hierarchical porous carbon derived from a chitosan/polyethylene glycol blend for high performance supercapacitors

**DOI:** 10.1039/c8ra00016f

**Published:** 2018-02-14

**Authors:** Yuerong Ba, Wei Pan, Shangchao Pi, Yaomin Zhao, Liwei Mi

**Affiliations:** School of Materials and Chemical Engineering, Zhongyuan University of Technology Zhengzhou 451191 PR China doctorpan0152@163.com; Center for Advanced Materials Research, Zhongyuan University of Technology Zhengzhou 451191 PR China mlwzzu@163.com

## Abstract

Nitrogen-doped hierarchical porous carbon (NHPC) materials were synthesized by using a chitosan/polyethylene glycol (PEG) blend as raw material through a facile carbonization–activation process. In this method, chitosan was used as a nitrogen-containing carbon precursor, low cost and large-scale commercial PEG was employed as a porogen. The physical and electrochemical properties of the resultant NHPC were affected by the ratio of chitosan and PEG. The sample obtained by the ratio of 3 : 2 exhibits a high specific surface area (2269 m^2^ g^−1^), moderate nitrogen doping (3.22 at%) and optimized pore structure. It exhibits a high specific capacitance of 356 F g^−1^ in 1 M H_2_SO_4_ and 271 F g^−1^ in 2 M KOH at a current density of 1 A g^−1^, and over 230 F g^−1^ can be still retained at a high current density of 20 A g^−1^ in both electrolytes. Additionally, the assembled symmetric supercapacitors show an excellent cycling stability with 94% (in 1 M H_2_SO_4_) and 97% (in 2 M KOH) retention after 10 000 cycles at 1 A g^−1^. These results indicate that the chitosan/PEG blend can act as a novel and appropriate precursor to prepare low-cost NHPC materials for high-performance supercapacitors.

## Abstract 2

NHPC was prepared from a low cost chitosan/PEG blend by a facile method for high performance supercapacitors.

## Graphical abstract

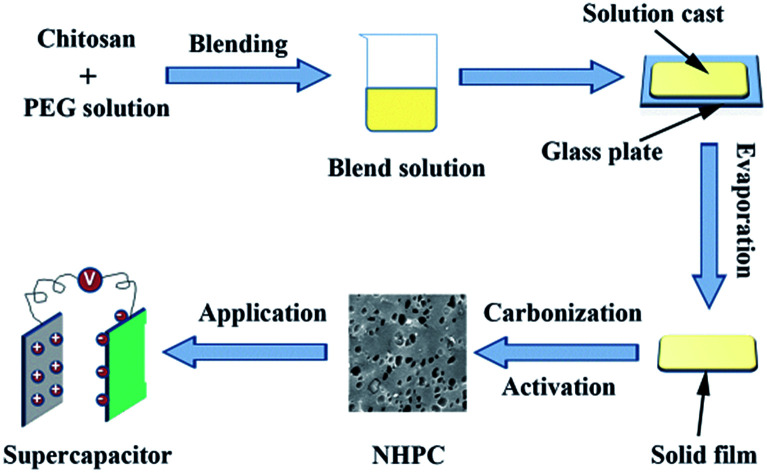

## Introduction

Supercapacitors, possessing a high power density, superior lifetime, fast charge–discharge ability and excellent safety property, have attracted great attention as next-generation energy storage devices.^1–6^ A variety of materials have been selected to build high performance supercapacitors to obtain high energy storage capability, such as carbonaceous materials, transition metal oxides and conducting polymers.^7–11^ Of them, carbon materials are thought to be appropriate electrode materials for supercapacitors due to many advantages such as low-cost, easy accessibility and excellent electrical conductivity.^12–15^ However, the relatively low specific capacitance is still hindering their use,^16^ so it is essential to develop carbon electrode materials with high specific capacitance.

In general, improvement of the specific capacitance of carbon materials is usually by introducing heteroatoms (*e.g.*, O, B, N) and regulating pore structure.^17–21^ Structural doping with heteroatoms, especially nitrogen, could improve the capacitance of carbon materials by introducing the pseudo-capacitance.^22–24^ In order to introduce nitrogen, postprocess, for example melamine immersion and ammonia heat treatment, is the most commonly used method.^25,26^ However, these methods may lead to a cumbersome process, pores structure collapse and unstable nitrogen functional groups under long-term/harsh working condition.^27,28^ In addition, the subsequently introduced nitrogen functional groups may block up the pores thus reducing the ion-accessible surface area.^25,29^ Although using nitrogen-enriched materials as precursors directly is able to overcome these weaknesses, they also exhibit some defects, such as uncontrollable pore structure and expensive template,^30,31^ which limits its performance optimization and large-scale applications. Considering these problems, synthesis of NHPC using the low-cost raw materials by a facile approach is very attractive.

Chitosan, the deacetylated derivative of chitin, is the second most popular natural polymer after cellulose.^32–34^ Because of a large number of amino-groups, chitosan can be used to synthesize N-doped carbon materials with excellent supercapacitor performance.^33,35^ However, in contrast to cellulose-based carbons, the research on the preparation of porous carbons using chitosan is still relatively few so far. There are only a few reports about the preparation of N-doped carbons from chitosan,^36,37^ and the pore structure of the obtained carbons is poorly developed, which would greatly hamper their application in supercapacitors.^38–40^ So adding appropriate substances to regulate pore structure is desired. PEG is a water soluble polymer, has good compatibility with chitosan, they can be mixed into homogeneous solutions.^41–43^ During the high temperature carbonization, PEG was removed by thermal degradation, the obtained samples possess ample pore structures, and the pore structure can be regulated by adjusting the ratio of chitosan and PEG. This provides us a good way to synthesize NHPC materials for high-performance supercapacitors and there are no other studies have been reported.

Herein, we used chitosan/PEG blend as raw materials to fabricate the NHPC by a facile method. It was found that the porous structure and specific capacitance of NHPC could be adjusted by changing the ratio of chitosan and PEG. The prepared NHPC exhibited an excellent electrochemical performance in both acidic and alkaline medium due to the high specific surface area and hierarchical porous structure.

## Experimental

### Preparation of NHPC

**Fig. 1:**
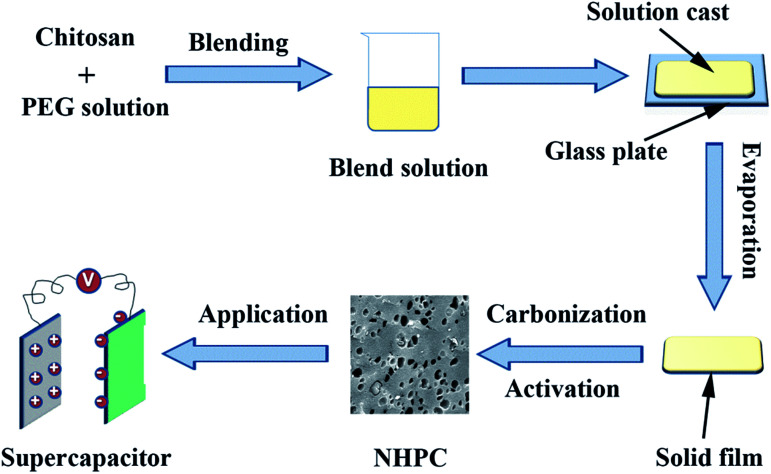
Schematic illustration of the preparation of NHPC materials.

Chitosan and PEG (*M*_w_ = 6000) with a total of 5 g were dissolved in 95 g acetic acid aqueous solution that was obtained by dissolving 2 g acetic acid in 93 g de-ionized water at 50 °C for 1.5 h with stirring to obtain a uniform solution. The solution was added onto a glass plate to form a solid film after the evaporation of solvent. Then the as-produced film was peeled off from the glass plate and carbonized at 800 °C under N_2_ atmosphere for 2 h with an increasing rate of 2 °C min^−1^. After cooled down to room temperature naturally, the carbonized film was ground into powder. Then the powder was blend with KOH solution with a power/KOH weight ratio of 1 : 3. The mixture was dried at 110 °C to evaporate the water. After that, the dried mixture was heated at 800 °C for 2 h at 5 °C min^−1^ under a N_2_ atmosphere before being cooled down to room temperature. The product obtained was washed with 1 M HCl solution and de-ionized water until a neutral pH. Finally, the washed powder was dried at 60 °C under vacuum overnight to get the NHPC. The sample was signified as the PEG-*x*%, where *x*% stands for the ratio of PEG/(PEG + CS). The synthesis process is schematically presented in Fig. 1.

### Characterization

The morphologies and component of the NHPC materials were characterized by a Zeiss Merlin Compact scanning electron microscope (SEM) equipped with an energy dispersive X-ray spectroscopy (EDX) system. High-resolution transmission electron microscopic (HRTEM) images were obtained with a JEOL JEM2100F microscope. The surface chemical properties of the samples were characterized by X-ray photoelectron spectroscopy (XPS, Escalab 250, USA). X-ray diffraction (XRD) patterns were conducted using a X-ray powder diffractometer (Bruker D8 Advance) with Cu-Kα irradiation. Raman spectra were acquired with a Raman spectrometer (LabRAM HR Evolution). Thermogravimetry (TG) was carried out on a TG209F1 under nitrogen flow. N_2_ adsorption–desorption isotherms were measured at 77 K using ASAP 2420 V2.09 A. The specific surface area was measured using the Brunauer–Emmett–Teller (BET) method and the pore size distribution (PSD) was determined using the classical Barrett–Joyner–Halenda (BJH) model.

### Electrochemical measurements

Working electrodes were prepared by pressing a disk (1 cm diameter) containing 80 wt% of active material, 10 wt% of acetylene black and 10 wt% of polytetrafluoroethylene onto nickel foam (used in KOH solution) and stainless steel mesh (used in H_2_SO_4_ solution). The active mass was 2.0–3.0 mg per electrode. The electrochemical performances of as-prepared electrodes were studied in 1 M H_2_SO_4_ and 2 M KOH solution using a three-electrode and two-electrode system on a CHI660E electrochemical workstation (Chenhua Instruments Co. Ltd., Shanghai). In the three-electrode system, platinum foil and Ag/AgCl electrode were used as the counter and reference electrodes, respectively. Cyclic voltammetry (CV), galvanostatic charge–discharge (GCD) and Electrical Impedance Spectroscopy (EIS) measurements were performed. The specific capacitance (*C*_g_) can be calculated by using the formula of *C*_s_ = *I* × Δ*t*/(*m* × Δ*V*), where *I* is the discharge current (A), Δ*t* is the discharge time (s), *m* is the mass (g) of the active materials, and Δ*V* is the potential window (V) during discharge.^44,45^ The specific capacitance of the two-electrode symmetrical supercapacitor cell can be calculated by using the formula of *C*_cell_ = *I* × Δ*t*/(*M* × Δ*V*), where *C*_cell_ is the total cell specific capacitance (F g^−1^), and *M* is the total mass (g) of active materials in both electrode. The energy and power densities (*E*, W h kg^−1^ and *P*, W kg^−1^) were calculated according to the equation of *E* = 1/2*C*_cell_*V*^2^ and *P* = *E*/*t*, in which *C*_cell_ is the specific capacitance of the device, *V* is the voltage decrease in discharge, and *t* is the discharge time. The long-term cycling performance of electrodes were measured by a symmetrical supercapacitor in 1 M H_2_SO_4_ and 2 M KOH solution at a current density of 1 A g^−1^ on a Land Battery Tester (LAND electronics Co. Ltd, Wuhan) at ambient temperature.

## Results and discussion

### Characterization of the prepared materials

**Fig. 2:**
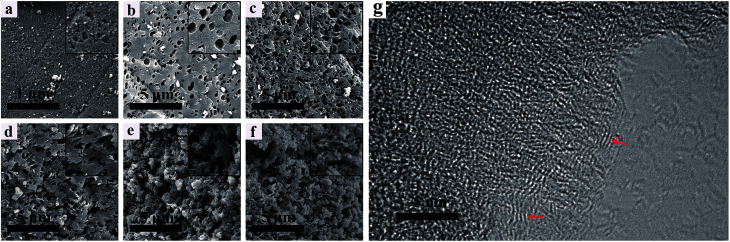
SEM images of (a) PEG-0%, (b) PEG-10%, (c) PEG-20%, (d) PEG-30%, (e) PEG-40%, (f) PEG-50% and (g) HRTEM image of the PEG-40% sample.

Fig. 2a–f show the SEM images of the NHPC materials. It can be seen that all samples possess pore structures, and such a porous structure renders the electrolyte able to penetrate into the bulk particles to form three-dimensional channels for ions' transportation. It is obviously observed that the microstructure and morphology are seriously affected by the PEG content. With the increase of PEG in the preparation process, the pores of samples are increase. In contrast, PEG-50% has the 3D network structure with plentiful and loose pores randomly. Fig. 2g shows the HRTEM images of PEG-40% samples. It can be seen that PEG-40% mainly exhibits an amorphous structure, and some locally ordered structure could be observed, as marked by the red arrows, indicating partial graphitization of the synthesized materials.

**Fig. 3:**
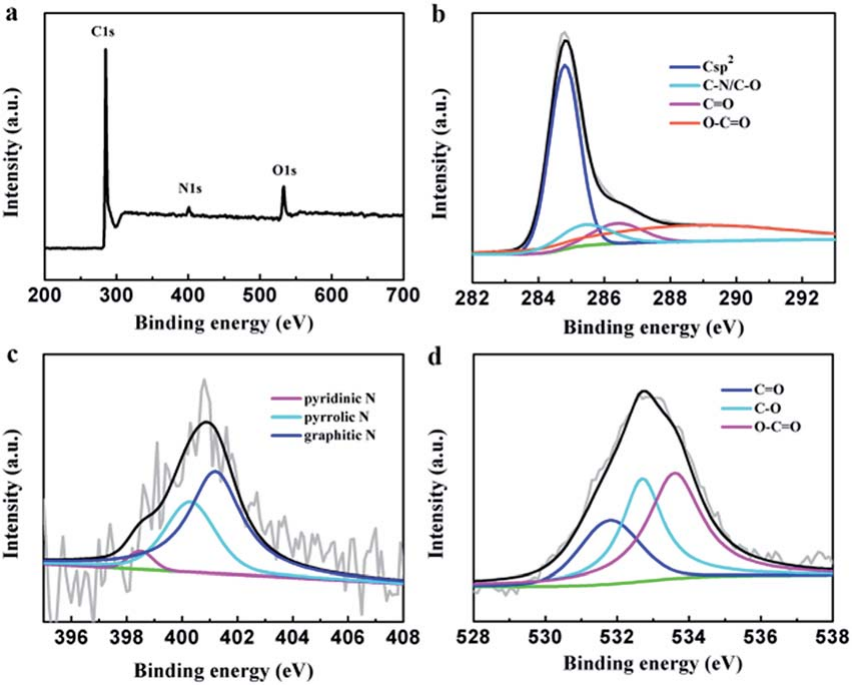
(a) XPS survey, (b) C 1s, (c) N 1s, and (d) O 1s of PEG-40%.

The element composition of the PEG-40% sample was determined by XPS measurement (Fig. 3). The strong signals in the survey XPS spectra reveal the existence of three peaks at 284.8, 400.9 and 532.6 eV, corresponding to C 1s, N 1s and O 1s, respectively (Fig. 3a). High resolution XPS measurements were performed to investigate the atom binding states. In case of C 1s, four peaks at 284.8, 285.4, 286.4 and 288.8 eV corresponding to Csp^2^, C–O/C–N, C=O and O–C=O groups was observed. The N 1s was determined by 398.5, 400.3 and 401.2 eV, which can be attributed to pyridinic N, pyrrolic N and graphitic N, respectively.^45^ In case of O 1s, three peaks at 531.8, 532.7, and 533.6 eV, corresponding to C=O (carbonyl), C–O (epoxy and hydroxyl), and O=C–O (carboxyl) groups are determined. The XPS results demonstrated the effective doping of nitrogen by chitosan in the preparation process.

**Fig. 4:**
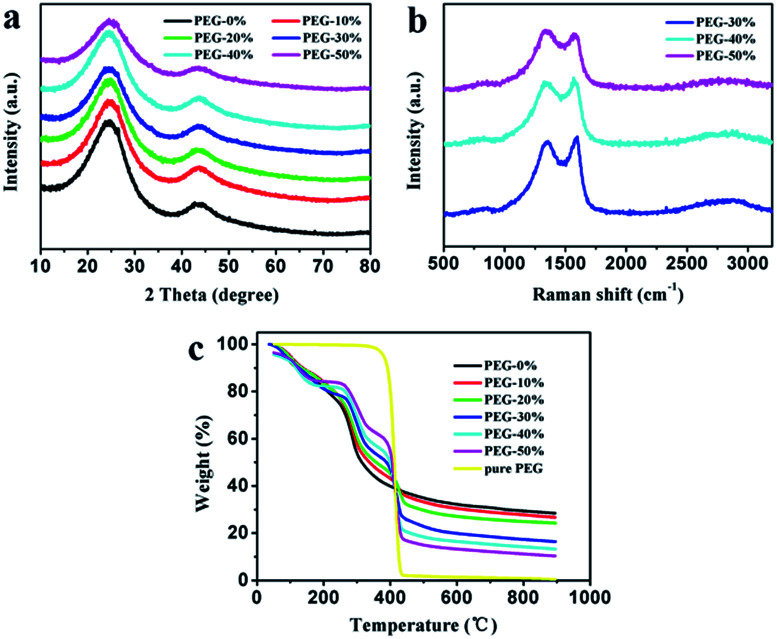
XRD patterns (a) and Raman spectra (b) of the carbon materials. Thermal decomposition curves (c) of different solid films.

The as-prepared carbon materials were further analyzed by XRD and Raman patterns to confirm the graphitized structure. All XRD patterns in Fig. 4a show the diffraction peaks at 2*θ* around 25° and 43°, corresponding to (002) and (100) crystallographic planes, respectively. From the picture it can be seen that PEG-50% had a low order degree, which is in good agreement with SEM analysis (Fig. 2). Fig. 4b presents the Raman patterns of PEG-30%, PEG-40% and PEG-50% samples. All three samples display G (around 1580 cm^−1^) and D (around 1340 cm^−1^) bands, reflection of ordered and disordered structures,^23^ respectively. And the narrower G-band and the lower intensity of *I*_D_/*I*_G_ often imply higher ordered structures and graphitizatio degree.^24,32,45^ The values of *I*_D_/*I*_G_ for PEG-30%, PEG-40% and PEG-50% were 0.93, 0.94 and 1.07, respectively, this result demonstrates that the degree of graphitization decreases with the PEG content increases, and PEG-50% sample possesses much lower degree of graphitization than others, which is identical with the results of above XRD analysis.

The thermal decomposition curves of PEG-0% to PEG-50% solid films and pure PEG were shown in Fig. 4c. The pure PEG exhibited only one stage around 360–440 °C due to the thermal degradation of PEG. When the temperature reaches to 800 °C, the weight loss of PEG was 99.2%, indicating that PEG was almost complete decomposition. The PEG-0% exhibited two stages around 80–150 °C and 250–350 °C due to the evaporation of water and thermal degradation of chitosan, respectively. The other samples showed three distinct stages from 30 to 900 °C, which were attributed to the evaporation of water (around 80–150 °C), thermal degradation of chitosan (around 250–350 °C), and thermal degradation of PEG (around 360–440 °C). It can be seen that carbon residue increases during following the increasement of chitosan, which is in good agreement with above analysis.

**Fig. 5:**
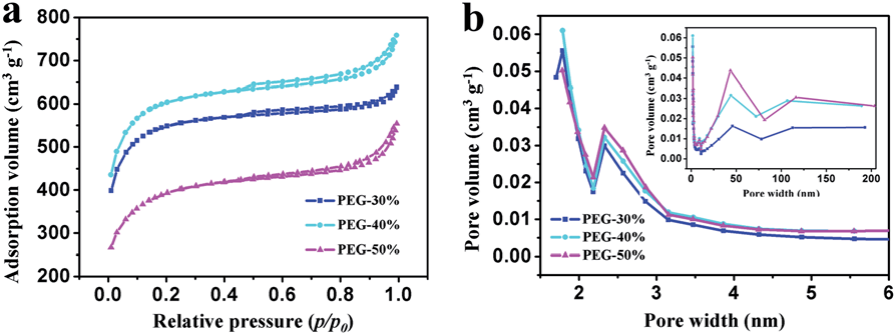
(a) N_2_ adsorption–desorption isotherms at 77 K and (b) PSD curves of PEG-30%, PEG-40% and PEG-50% samples.

N_2_ adsorption–desorption isotherms were performed to examine the specific surface area and the pore size and distribution in the samples. As shown in Fig. 5a, all isotherms exhibit type IV with uptake at low pressure and small hysteresis loops ranging from 0.5 to 1.0, which imply the coexistence of micropores, mesopores, and macropores in these samples.^8^ This result can be further confirmed by using BJH model to calculate the pore size distribution. And the hierarchical porous structure is conform to the ideal supercapacitor electrode materials that containing macropores, mesopores and micropores for ion buffering reservoir, ion transport and enhancement of charge storage,^33^ respectively. The data on specific surface area and pore volume are listed in Table 1. With the increase of PEG content, the total pore volume was increase, moreover, the mesopores and macropores volume was increase and the micropores volume was decrease. Compared with PEG-40%, the total pore volume of PEG-50% has a slight increase due to the high content of PEG. When the content of PEG is too high, a large number of PEG gather together and leave large pores after decomposition, and large pore size may lead to pores structure collapse seriously in the process of post treatment and more pore volume was destroyed when grinding into powder. With the increase of PEG content, the specific surface area increased firstly and then decreased, this is the results of combined action of pore volume and pore size. With the increase of PEG content, the pore volume was increase, although the pore size would also increase, the pore volume increased substantially, so the specific surface area will increase. The pore volume increased little when the PEG content reaches a certain value, the effect less than the pore size, therefore the specific surface area decreased rapidly with the increase of PEG content. From the above results, it can be seen that different proportion can influence the specific surface area and pore volume. Sample PEG-40% has the most suitable pore size and size distribution, which is expected to enhance its behavior as a capacitor.

**Table 1:** Porous property of PEG-30%, PEG-40% and PEG-50% samples

Sample	*S*_BET_ (m^2^ g^−1^)	*V*_total_ (cm^3^ g^−1^)	*V*_micro_	*V*_meso_	*V*_macro_	Pore size (nm)
PEG-30%	2052	0.429	0.146	0.226	0.057	2.7
PEG-40%	2269	0.516	0.107	0.302	0.107	3.5
PEG-50%	1436	0.520	0.091	0.309	0.120	3.6

### Electrochemical performance

**Fig. 6:**
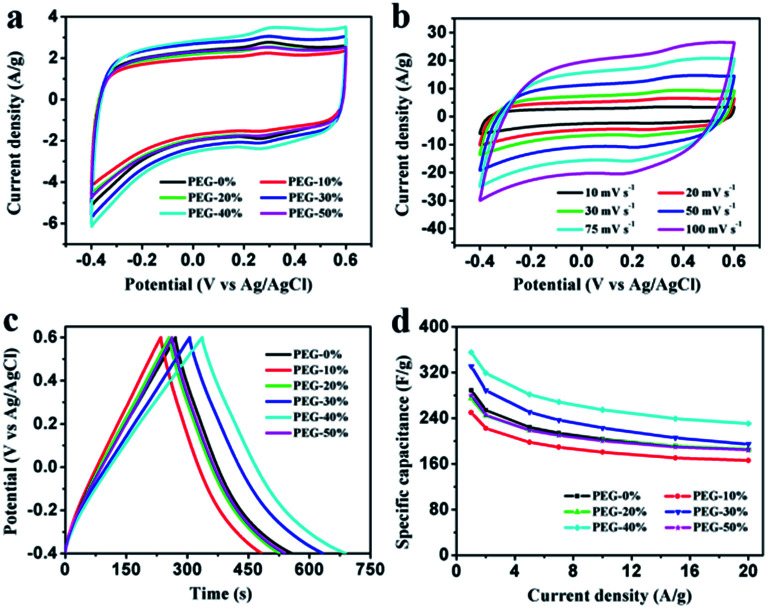
Electrochemical performances of the PEG-*x*% materials based electrode measure in a three-electrode system in 1 M H_2_SO_4_ aqueous electrolyte. (a) CV curves at a scan rate of 10 mV s^−1^; (b) CV curves of PEG-40% electrode material at different scan rates; (c) GCD curves at a current density of 1 A g^−1^; (d) specific capacitances at different current densities.

**Fig. 7:**
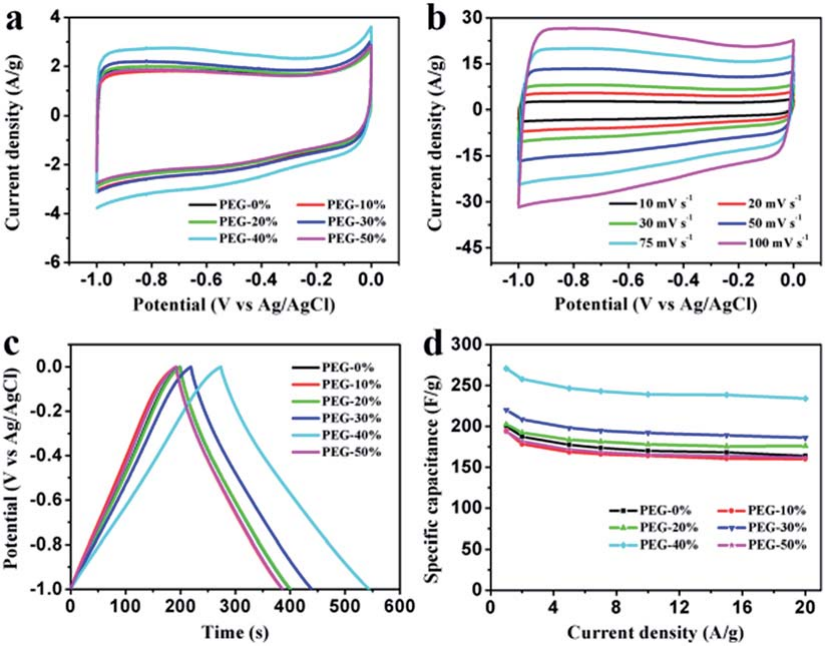
Electrochemical performances of the PEG-*x*% materials based electrode measure in a three-electrode system in 2 M KOH aqueous electrolyte. (a) CV curves at a scan rate of 10 mV s^−1^; (b) CV curves of PEG-40% electrode material at different scan rates; (c) GCD curves at a current density of 1 A g^−1^; (d) specific capacitances at different current densities.

Electrochemical properties of the resultant NHPC materials were measured using a standard three-electrode configuration in both acidic and alkaline solution. Fig. 6a and 7a show the typical CV curves of samples at a scan rate of 10 mV s^−1^ in 1 M H_2_SO_4_ and 2 M KOH, respectively. In both cases, the covered area of PEG-40% sample is larger than the other samples, it illustrates that PEG-40% sample possesses the highest capacitance, which was consistent with the physical characteristics. And all the samples present a rectangular-like shape with obvious redox peaks due to the introduction of N heteroatom in carbon materials. The pseudocapacitance in acidic electrolyte is more obvious than that in basic electrolyte, probably owing to the Lewis base behavior of the nitrogen functionalities in the carbons.^44^ In order to further investigate the capacitance behavior of PEG-40% material, the CV curves at different scanning rates were measured. The shapes of CV curves can be well retained even under the high scan rate (Fig. 6b and 7b), indicating a superb capacitive behavior.

Fig. 6c and 7c show the GCD curves of each sample at the current density of 1 A g^−1^. The curves show a typical triangular shape with slight curvature, and the deviation from linearity is due to the pseudo faradic reactions during the charging–discharging process, which is consistent with the CV curves. Compared with other samples, PEG-40% has the much larger capacitance owing to its high specific surface area (2269 m^2^ g^−1^) and moderate N-doped (3.22% obtained by EDX). Its *C*_s_ is as high as 356 and 271 F g^−1^ in 1 M H_2_SO_4_ and 2 M KOH, respectively. Fig. 6d and 7d show the capacitance retention for current density from 1 to 20 A g^−1^, the specific capacitances progressively decrease with the increase of current density due to the increasing diffusion limitation. However, PEG-40% can still retain over 230 F g^−1^ at a high current density of 20 A g^−1^ in both electrolytes, which means a good rate performance for the supercapacitor. The performances of different N-doped porous carbons are listed in Table 2. Overall, the performance of the as-prepared carbon materials in this work is superior to those proposed in the literature.

**Table 2:** Comparison of performances of different N-doped porous carbons

Materials	*S*_BET_ (m^2^ g^−1^)	*C*_g_ (F g^−1^)	Cycle performance	Reference
K800	2435	197 (0.2 A g^−1^)	92% after 10 000 cycles at 1 A g^−1^	6
RGH_3_-1	173	258 (0.1 A g^−1^)	97% after 4000 cycles at 1 A g^−1^	16
N-PGCNS_0.2–1–2_	2130	337 (0.5 A g^−1^)	88% after 5000 cycles at 2 A g^−1^	24
NCNF3	763	251 (0.1 A g^−1^)	99% after 2000 cycles at 5 A g^−1^	25
NHPC-800	1542	242 (0.2 A g^−1^)	94% after 10 000 cycles at 1 A g^−1^	32
3DHCG	1511	320 (1 A g^−1^)	96% after 2000 cycles at 2 A g^−1^	45
PEG-40%	2269	356 (1 A g^−1^)	94% after 10 000 cycles at 1 A g^−1^	This work
		271 (1 A g^−1^)	97% after 10 000 cycles at 1 A g^−1^	This work

**Fig. 8:**
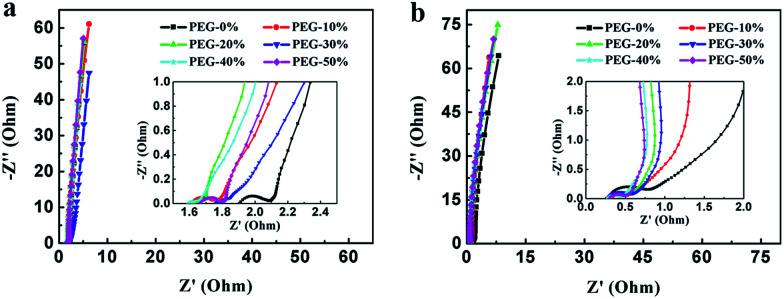
Nyquist plots measured in 1 M H_2_SO_4_ (a) and 2 M KOH (b). The inset shows a magnified view of the high frequency region of the impedance spectra.

Fig. 8 shows the Nyquist plot of the samples at the frequency range from 0.01 Hz to 100 kHz to further investigate the capacitive property of the electrodes. The Nyquist plots were measured in a three-electrode system at the open circuit voltage. And the plot of PEG-40% is modeled and interpreted with the assistant of an appropriate electric equivalent circuit. The Nyquist plot could be divided to three parts, including an uncompleted semicircle part at high frequency, an inclined portion about 45° at the middle frequency and a linear part at low frequency. The high-frequency intercept with the real axis of PEG-40% gives the resistance value (*R*_s_) of 1.6 Ω (in 1 M H_2_SO_4_) and 0.29 Ω (in 2 M KOH) which includes the electrolyte resistance, the active material resistance, and the active material interface resistance. The lower value of *R*_s_ is associated with better electrode conductivity. The diameter of the semi-circle is a charge-transfer resistance (*R*_ct_), which is attributed to the charge transfer at the interface of the electrode and electrolyte, and a smaller semicircle means smaller charge transfer resistance. The *R*_ct_ of PEG-40% electrode is 0.03 Ω (in 1 M H_2_SO_4_) and 0.13 Ω (in 2 M KOH), which is lower than those of other electrode materials. The 45° slope region at middle frequency can be attributed to the ions diffusion/transport from the electrolyte to the pore on the surface of samples. The short lengths of these slopes indicate that the electrolyte ions diffuse fast in this carbon framework. The almost vertical line represents the dominance of ideal double-layer charge/discharge behaviors at low frequencies.

**Fig. 9:**
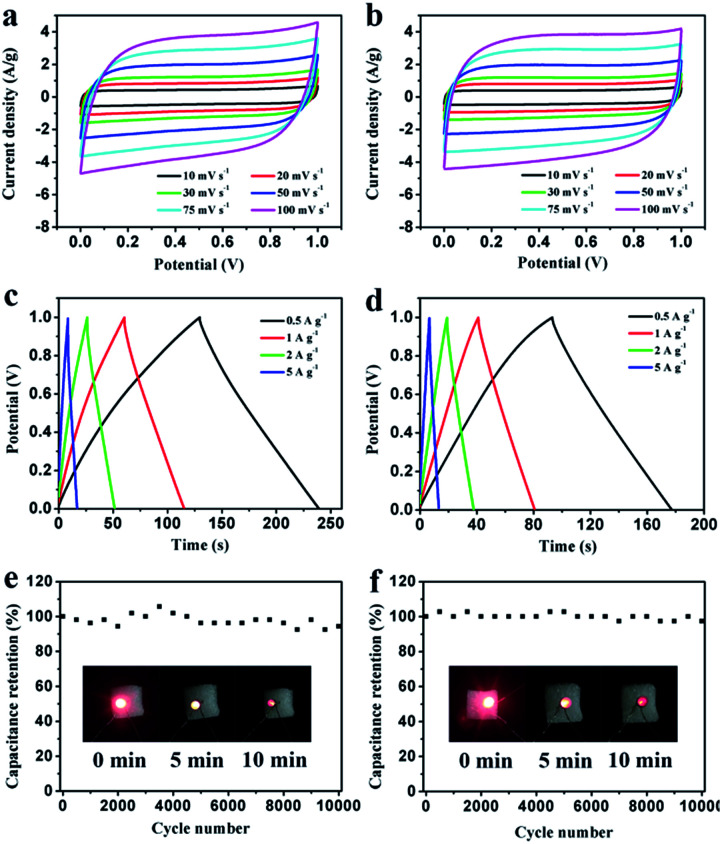
Electrochemical performance of PEG-40% symmetrical supercapacitors in 1 M H_2_SO_4_ and 2 M KOH, respectively. (a, b) CV curves at different scanning rates; (c, d) GCD curves at different current densities; (e, f) cycling stability at a 1 A g^−1^.

To further study the electrochemical behavior of the PEG-40% electrode as a real capacitor in 1 M H_2_SO_4_ and 2 M KOH solution, a symmetrical two-electrode configuration was constructed. It can be observed that the fabricated symmetric supercapacitor shows near-rectangular shapes in both acid and basic solution (Fig. 9a and b), even at high scan rates, indicating its ideal capacitive behavior.^45^ Moreover, the GCD curves in Fig. 9c and d display good symmetrical linear curves, revealing its high electrochemical stability. The specific capacitance of the symmetric supercapacitors calculated from GCD curves is 55 F g^−1^ and 42 F g^−1^ (220 F g^−1^ and 168 F g^−1^ for single electrode) at 0.5 A g^−1^, 91% and 86% capacitance is retained even at 5 A g^−1^ in 1 M H_2_SO_4_ and 2 M KOH, respectively. Stability testing was conducted under 1 A g^−1^ for 10 000 cycles. Fig. 9e and f show the cycling stability of the symmetric supercapacitors in 1 M H_2_SO_4_ and 2 M KOH, respectively. The capacitance retention is about 94% and 97% of its initial value after 10 000 cycles, indicating the excellent cycling stability of PEG-40%. To further demonstrate the device performance after cycles, two supercapacitors were connected in series to light a red LED. As shown in Fig. 9e and f, after charging to 2.2 V at a current density of 1 A g^−1^, the device could power a red LED, and even after 10 min the red LED still remained bright, indicating its excellent electrochemical properties even after 10 000 cycles.

**Fig. 10:**
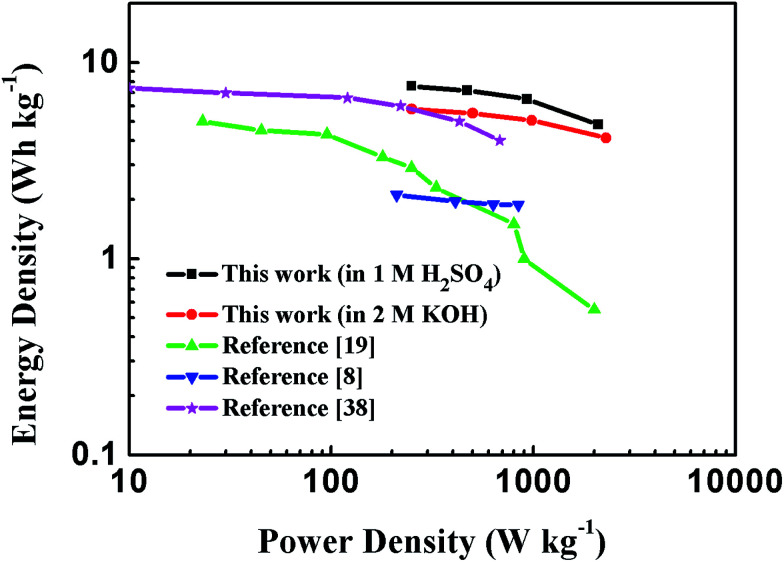
Ragone plot comparing the energy density and power density of PEG-40% symmetric supercapacitor with other reported carbon materials in an aqueous electrolyte.

Energy and power densities are key parameters to evaluate the performance of a certain material when applied as electrodes for supercapacitors. The Ragone plot of PEG-40% symmetric supercapacitor in 1 M H_2_SO_4_ and 2 M KOH aqueous solutions calculated from discharge curves at different current densities is displayed in Fig. 10, as well as its comparison with the representative porous carbon-based supercapacitors. The results showed that the energy density of the supercapacitors utilizing PEG-40% as electrode material could reach 7.58 W h kg^−1^ with a corresponding specific power density of 0.25 kW kg^−1^ in 1 M H_2_SO_4_ solution, and an energy density of 5.79 W h kg^−1^ could be obtained with 0.25 kW kg^−1^ of specific power in 2 M KOH electrolyte, which is higher than those of commercially available supercapacitors (3–5 W h kg^−1^)^24^ and most carbon-based/N-doped carbonaceous aqueous capacitors reported in previous articles.

## Conclusions

In summary, NHPC materials have been fabricated by the carbonization of chitosan/PEG blend and activation with KOH. Benefiting from its hierarchical porous structure and numerous pseudocapacitive functional groups, the carbon materials exhibits an excellent performance. As an example, the PEG-40% sample has micro, meso-, and macro- hierarchical porous structure and possesses a high specific surface area of 2269 m^2^ g^−1^. It can deliver high specific capacitances of 356 F g^−1^ (in 1 M H_2_SO_4_) and 271 F g^−1^ (in 2 M KOH) at the current density of 1 A g^−1^, retain 65% and 86% at 20 A g^−1^. Furthermore, the assembled symmetric supercapacitors show an excellent cycling stability with 94% (in 1 M H_2_SO_4_) and 97% (in 2 M KOH) retention after 10 000 cycles at 1 A g^−1^. The outstanding capacitive behavior is attributed to the unique features including a high specific surface area, reasonable pore size and pore size distribution, and moderate nitrogen doping. These demonstrated that using low-cost biopolymer as the raw material to produce NHPC materials is a promising approach for the development of energy storage systems.

## Conflicts of interest

There are no conflicts to declare.

## Supplementary Material
